# Multidisciplinary Perspectives of Clinical Trials in Theranostics

**DOI:** 10.1055/s-0045-1812102

**Published:** 2025-10-07

**Authors:** Kunthi Pathmaraj, Sze Ting Lee

**Affiliations:** 1 Department of Molecular Imaging and Therapy, Austin Health, Victoria, Australia; 2 School of Health and Biomedicine, RMIT University, Melbourne, Australia; 3 Olivia Newton-John Cancer Research Institute, Melbourne, Australia; 4 School of Cancer Medicine, La Trobe University, Melbourne, Melbourne, Australia; 5 Department of Medicine and Surgery, The University of Melbourne, Melbourne, Australia

**Keywords:** clinical trials, clinical trials networks, Good Clinical Practice, radiation safety, theranostics

## Abstract

Theranostics and clinical trials are driving the future of nuclear medicine and require a multidisciplinary approach to achieve the best possible care for the patient. Theranostics refers to using chemical compounds with similar diagnostic and radiotherapeutic applications. Therefore, the biodistribution remains the same for the imaging and therapy portions, a low radiation dose being delivered during imaging, and a higher radiation dose being delivered to the target disease areas during the treatment phase. The new era of theranostics has revolutionized nuclear medicine through the prospective phase 3 clinical trials, which have resulted in its adoption into the treatment paradigms of patients, particularly those with neuroendocrine tumors and prostate cancer. Molecular imaging can be used to assess the role and utility of new tracers and molecular endpoints to help improve the understanding of tumor biology and evaluation of treatment response, leading to intelligent clinical trial design and more rapid drug development. Clinical trials networks such as EANM Research Ltd. (EARL), SNMMI Clinical Trials Network (CTN), and Australasian Radiopharmaceutical Trials Network (ARTnet), encourage a standardized approach and promote a collaborative approach to clinical trials in molecular imaging. Clinical Trials in Theranostics require the skills and expertise of a multidisciplinary team including the principal investigator, nuclear medicine specialists, study coordinators, medical physicists, radiopharmaceutical scientists, nuclear medicine technologists, research nurses, and research assistants. All personnel involved in theranostics clinical trials should be certified in Good Clinical Practice (GCP). Accurate documentation and record keeping in clinical trials provides validation that clinical trials is conducted at the highest ethical and clinical standards, meets the expectation of the study protocol, and adheres to GCP. Radiation safety is a critical factor for staff, patients, and the public in theranostics and clinical trials and must follow country-specific guidelines and international guidelines to ensure basic safety standards are met. As theranostics and clinical trials continue to stamp their mark in molecular imaging and radionuclide therapy, it is imperative that nuclear medicine professionals remain upskilled and adequately trained in these two aspects of the profession to ensure optimal care is delivered to the patient.

## Introduction

Theranostics is another step forward in the application of radionuclide therapy, referring to using a similar chemical compounds with similar diagnostic and radiotherapeutic applications. Therefore, the biodistribution remains the same for the imaging and therapy portions, a low radiation dose being delivered during imaging, and a higher radiation dose being delivered to the target disease areas during the treatment phase.

Theranostics is not a new concept in nuclear medicine. I-131 for thyroid cancer diagnosis and treatment can be considered “old era” theranostics. 1 Ga-68 prostate-specific membrane antigen (PSMA) and Lu-177 PSMA can be considered “new era” theranostic. 2 The new era of theranostics has revolutionized nuclear medicine through the prospective phase 3 clinical trials, which have resulted in its adoption into the treatment paradigms of patients, particularly those with neuroendocrine tumors and prostate cancer. Clinical trials can be single-site trials or multicenter trials and are conducted in various settings such as industry-sponsored trials, or as part of a collaborative group/investigator-initiated trials. As theranostics evolves and gathers momentum in the nuclear medicine community, it has become imperative that nuclear medicine professionals are skilled and trained for their role in theranostics and clinical trials.

Molecular imaging, including general nuclear medicine (planar and single-photon emission computed tomography [SPECT]) and positron emission tomography (PET) imaging, has a key role in clinical trials. Molecular diagnostic imaging can be used to assess the role and utility of new tracers and molecular endpoints to help improve the understanding of tumor biology and evaluation of treatment response, leading to intelligent clinical trial design and more rapid drug development. This has led to the concept of theranostics being patient focused—to select the right patient for the right treatment at the right time.

In this article, we describe the multidisciplinary requirements for conducting clinical trials in theranostics, highlighting opportunities and challenges for nuclear medicine departments.

## Clinical Trials

Clinical trials are research oriented, study new treatments and evaluate their effects on human health outcomes. 3 There are four phases of biomedical clinical trials that nuclear medicine departments often participate in. 3 Phase I studies usually test new drugs for the first time in a small group of participants to evaluate a safe dosage range, toxicity, and biological aspects such as pharmacokinetics, biodistribution, and radiation dosimetry. Phase I studies are important for understanding that radiation exposure is within acceptable limits for the intended diagnostic or therapeutic application, and that the properties of the injected radiopharmaceutical are suitable for further clinical trials. These studies maybe best conducted at sites with relevant expertise and equipment for such trials. Phase II studies test treatments that have been found to be safe in phase I, and explore the efficacy of the study drug. Phase II trials also continue to assess the safety of the treatment in a larger patient population than phase 1 studies and hence maybe better suited for nuclear medicine departments that have a large patient catchment for the relevant clinical indications of the study objective. Phase III studies are conducted on larger populations, and may be conducted in different regions/countries, and generate evidence necessary for potential approval. Phase III trials may be more widely accessible depending on the patient group being evaluated and hence a wider range of nuclear medicine departments may be able to participate in phase III trials. Phase IV studies take place postapproval and are to monitor efficacy and safety in larger patient populations and long timeframes.

Theranostics trials such as TheraP, VISION, PSMAFore, ENZAp, and UpFront are recent examples of theranostic clinical trials that have practice changing and/or have led to health technology assessments/funding for a specific clinical indication (prostate cancer). There are many clinical trials currently underway exploring theranostic treatments in a broad range of cancer indications. 4 5

Conducting clinical trials, which incorporate radiopharmaceuticals for imaging, especially multicenter trials, requires careful organization and attention to detail. Radiopharmaceutical manufacturing may be onsite or reliant on a centralized radiopharmacy. Site variations in PET, SPECT, and bone mineral density (BMD) scanners will occur for imaging protocols, image reconstruction, data analysis, and image display. There is a reconstruction variability even between centers utilizing similar scanners. These types of variations will add a complexity in reporting the scans at the central reading site, for example, interpreting standardized uptake values (SUVs) that are not standardized across different scanners. Data integrity and confidentiality and quality assurance of data transmission are other aspects in clinical trials that need to be monitored as per the Good Clinical Practice (GCP) guidelines. 6 7 Therefore, it is necessary to design a standardized approach to the conduct of clinical trials.

Clinical trials are usually sponsored by pharmaceutical companies, and these industry-led clinical trials are primarily aimed at developing new drugs that ultimately gain regulatory approval and can include activities or services in a nuclear medicine department. However, clinical trials can also be investigator-initiated trials, usually developed by academic investigators or cooperative trial groups, such as radiation oncology, hematology, and oncology to address a clinical need.

Clinical trial designs must include robust imaging protocols and methodologies that are standardized, which can be translated into routine theranostics studies to assist with adopting a standardized approach to reporting of clinical trial scans. Multicenter clinical trials require scanner uniformity, stability of radiopharmaceuticals, and consistency of imaging data to allow comparability across sites. This would include optimizing and standardizing clinical protocols and imaging protocols (such as injected radioactivity, administration technique, blood sugar level, acquisition parameters, reconstruction parameters, image analysis, data handling, and picture/data archiving) in the setting of clinical trials to ensure the scanner (PET/CT or SPECT/CT) is performing to specified and accepted standards. It is important that data from scanners across multiple sites (national and international) can be used in a meaningful manner by central reading sites for both qualitative and quantitative assessment of clinical trial data. The role of clinical trials networks in achieving this, such as outlined below, is essential to ensure that standardized acquisition and reconstruction protocols and image analysis methods are performed in multicenter trials. This ensures quantitative endpoints are not affected by variability in imaging, data reconstruction, image analysis, and reporting of trial scans.

## Clinical Trials Networks

Initiatives are being undertaken by nuclear medicine societies worldwide to establish clinical trials networks. The purpose of such bodies is to provide quality assurance of both imaging and radiopharmaceutical manufacturing, enhance the comparability of data acquired by molecular imaging, and encourage a standardized approach to clinical trials in molecular imaging.

### European Association of Nuclear Medicine Research Ltd.

European Association of Nuclear Medicine Research Ltd. (EARL) was established in 2006, by the EANM as an initiative to promote multicenter nuclear medicine and research, to enhance the comparability of data acquired by molecular imaging, and accredit PET/CT scanners, thereby facilitating standardized PET/CT imaging. 8 EARL was able to show that gross PET/CT calibration errors can be identified and longitudinal variability in PET/CT performances can be reduced. The EARL program uses a specific set of quality control (QC) measurements. 9 The first QC measurement, calibration QC, is designed to verify the basic calibration of the PET/CT scanner relative to the dose calibrator that is used to measure the dispensed FDG (fludeoxyglucose) radioactivity for patients undergoing PET scans. The second QC measurement, image quality QC, uses the NEMA NU 2 image quality phantom to calculate the reconstruction settings that results in comparable SUVs across different scanners by harmonizing SUV recoveries and leading closer to expected SUVs.

### SNMMI CTN

The Clinical Trials Network (CTN) was formed in 2008 by the Society of Nuclear Medicine and Molecular Imaging (SNMMI) to help facilitate the effective use of molecular imaging radiopharmaceuticals in clinical trials and to facilitate the effective use of molecular imaging in clinical trials through standardization, coordination, and education for drug development and regulatory approval. 10 The CTN is playing a valuable role in promoting the need for better standardization of methods when PET/CT is used for quantitative purposes in clinical trials. CTN also facilitates successful radionuclide-specific SPECT/CT scanner calibration, which is critical for accurate dosimetry measurements in theranostics, where posttreatment SPECT scanning is increasingly utilized, as in the instances of patients being treated with Lu-177-based radionuclide therapy. SNMMI launched the Therapy CTN (TCTN) in June 2023 in light of the rapid growth of radiopharmaceutical therapy-based research trials. 11

### Australasian Radiopharmaceutical Trials Network

Australasian Radiopharmaceutical Trials Network (ARTnet) was established in 2014 as a joint venture between the Australian and New Zealand Society of Nuclear Medicine (ANZSNM) and the Australasian Association of Nuclear Medicine Specialists (AANMS) with a view to promote and facilitate innovative collaborative clinical research utilizing radiopharmaceuticals for imaging or therapy. 12 Certification of sites, protocol review, and standardization of imaging procedures are important services provided by ARTnet, along with helping develop investigator brochure manual outlining radiopharmaceutical synthesis method and QC, development of validation QC worksheets, and trial patient-specific worksheets. A rather unique process that ARTnet facilitates as a clinical trial network is central image review for multicenter clinical trials, which ensures standardized reporting necessary for phase II and III trials.

The ARTnet site initiation program utilizes the ARTnet phantom to document PET or SPECT camera performance for clinical trials. The ARTnet phantom assessment can be used for either a “general” assessment of an imaging device (SPECT or PET camera) to document basic performance under standard conditions, not attached to any particular trial, or a “specific” assessment designed to determine the suitability of imaging equipment for an individual clinical trial. 13 14

In summary, clinical trials networks such as those mentioned above are playing an important role in standardizing and maintaining consistency in radiopharmaceutical production, QC, and imaging so that molecular imaging can be confidently, reliably, and accurately used in the setting of clinical trials and theranostics.

## Multidisciplinary Requirements for Clinical Trials

*Principalinvestigator* (PI) is responsible for the conduct of a clinical trial in accordance with the approved clinical trial protocol. 15 The PI may be a senior personnel from the nuclear medicine department, or from a collaborative referring department, such as surgery, medical oncology, radiation oncologist, endocrinologist, etc. Some duties of the PI usually include:

- Ensuring that appropriate approvals are obtained prior to the commencement of the trial
- Ensuring any contractual requirements such as those under a clinical trial agreement
- Complying with the requirements for consent as approved in the trial protocol
- Ensuring participants' welfare during the clinical trial
- Provide the necessary clinical care to study participants required, including managing adverse events
- Providing reports to the local Research Governance Office (RGO)
- Providing site information to the sponsor or Coordinating Principal Investigator (CPI) for annual progress reports and the final report to the Human Research Ethics Committee (HREC) and the site
- Disclosing and managing actual, potential, or perceived conflicts of interest
- Retaining clear, accurate, secure, and complete records of all clinical trial documentation including clinical trial data and primary materials
- Complying with the applicable regulatory requirement(s) related to the reporting of unexpected serious adverse drug reactions to the regulatory authorities and the sponsors. Reporting suspected breaches of the code to the relevant institution and/or authority
- Supervising and working with site study coordinator/research nurses/nuclear medicine technologists (NMTs) for the management Clinical Trials.

The tasks of the NMT, research nurses, research assistants, and trial coordinators in clinical trials and theranostics can often intersect depending on the nuclear medicine department, the staffing available for clinical trials, and can also be country specific. It is also dependent on the level of multidisciplinary support staff available in a particular department. The tasks entrusted to NMTs can be determined by their qualification, expertise, skillset, and experience. In some instances, the scope for advanced practice that is available at a given site/given country.

*Clinicaltrialsadministration* can be undertaken by study coordinators, technologists, research nurses, or research assistants and can include the following 16 :

- review of clinical trial agreement and pro forma in conjunction with study investigators, research coordinators, and data managers
- completion of trial-mandated training and credentialing activities
- signing and lodgment of delegation logs
- participating in site-specific assessments
- assessing protocol for scheduling, imaging, and phantom requirements
- imaging and submitting phantoms to sponsor/monitor
- completing training sessions by sponsor
- maintaining GCP certification and applying GCP for trial-related activities
- scheduling trial patients appropriately among a clinical workload, ensuring that multiple imaging time points are booked correctly, scan uptake times are compliant with the trial protocol, and that enough time is allocated to complete the imaging requirements
- liaising with trial coordinators to book scans and ensuring correct patient preparation sent to patient
- informed consent from the patients for clinical trials is usually obtained by the PI or the delegated nuclear medicine physician/research assistant

*Instrumentation and scanner QC* may be performed by NMTs or medical physicists and can include 17 :

- performance of baseline and interval QC testing of scanners as stipulated by trial protocols
- data analysis, extraction, and export to required location
- follow-up and resolution of queries
- As a general rule, nuclear medicine imaging sites taking part in clinical trials should perform PET/CT and SPECT/CT phantom acquisitions as part of QC requirements that are stipulated by the clinical protocol and through a recognized trials network such as EARL, CTN, or ARTnet

*Radiopharmaceutical production* may be received from a vendor, central radiopharmacy, or produced in-house by appropriately trained NMT/radiopharmaceutical scientist. 16

- In-house radiopharmaceuticals production should be manufactured by staff appropriately trained in both radiopharmaceutical manufacturing and QC testing, and performed in an appropriate equipment and environment. Good radiopharmaceutical practice should be adopted wherever possible for continued process control and continually striving for high-quality standards. Where applicable, radiopharmaceuticals should be prepared according to regulatory and monograph guidelines. A documented procedure (e.g., standard operating procedure [SOP] or work instruction) describing the entire process for manufacture specific to the infrastructure onsite should be available and staff training and compliance to this procedure should be recorded, as per the SOP.
- Radiopharmaceuticals, which are produced centrally or received from a vendor, must have been appropriately transported with a valid certificate of analysis to ensure that the product is safe to be injected into patients. This has to be received and documented prior to administration.

*Patient-related duties* are usually performed by an NMT or nurse and can include:

- collection of patient information via questionnaire
- patient interview
- intravenous cannulation of the patient for tracer administration
- taking blood samples (if, and when required)
- administering the radiopharmaceutical
- patient positioning on the scanner and scanning as per study protocol
- completion of technical data sheets to gather information about the scan that will be required for reporting
- NMTs involved with clinical trials should follow the protocol required for scan acquisition requirements.

Informed consent from the patients for clinical trials is usually obtained by the PI, or the appropriate person in the delegation log.

*Postscan administrative duties* may be performed by NMTs, trial coordinators, nurses, or research assistants and can include:

- data analysis and generation of results
- uploading images remotely to central reading sites
- local record keeping
- archiving images and relevant information pertaining to the study
- documenting any modifications to scan protocol, including protocol violations
- follow-up and resolution of queries from study investigators

**Fig. 1:**
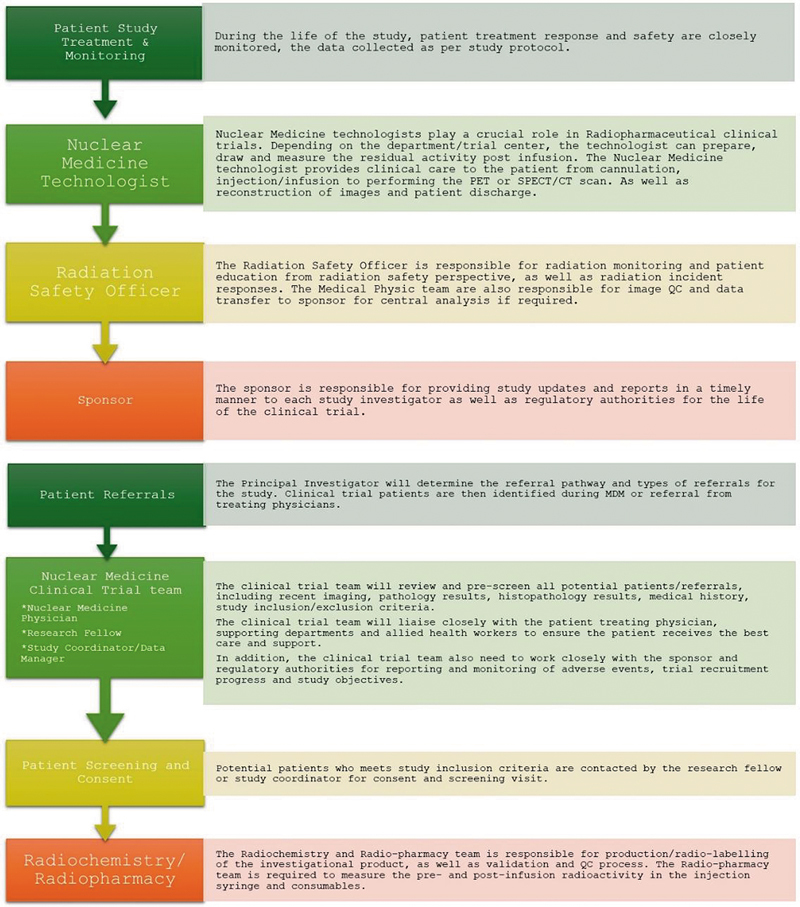
Roles and responsibilities of multi disciplines involved in Theranostics clinical trials.

The roles and activities of the abovementioned multidisciplines in the conduct of clinical trials is summarized in a tailored flowchart ( Fig. 1 ).

## Documentation and Record Keeping in Clinical Trials

Accurate documentation and record keeping in clinical trials provides validation that clinical trials is conducted at the highest ethical and clinical standards, meets the expectation of the study protocol, and adheres to GCP. 7 There are a wide range of documents encountered in clinical trials, namely, study-specific protocols, imaging manuals, laboratory manual, pathology manuals, trial agreements, case report forms, trial-specific worksheets, technical data sheets, recording of adverse events, data transmittal forms and records, archiving (both physical and electronic) delegation logs, and training/credentialing logs. GCP should always be observed with documentation and record keeping. Deviation should be avoided wherever possible to avoid exclusion of results from that trial. Unavoidable protocol deviations should be documented in detail and communicated in a timely manner to the PI and the sponsor of the clinical trial. Good documentation will permit audit trails to be conducted with ease.

## Radiation Safety

Patients who receive all administered radiopharmaceuticals, for both diagnostic and therapeutic purposes, must be appropriately educated and consented for the radiation protection required for the radiopharmaceutical. Administration of the radiopharmaceutical should be performed by skilled staff, using appropriate personal protective equipment such as gowns, gloves, and suitable eye goggles, and employing lead shields as appropriate. Most radiopharmaceuticals are excreted either via the gastrointestinal or renal systems, and hence radiation spill kits must be readily available in the department to manage spills. 17 Usually, patients are required to remain in the department for a prescribed period of time relevant for the radiopharmaceutical, for purposes of observation, and for patients to reach the recommended radiation exposure/emissions level before they can be discharged to the public. The recommended level for discharge will be guided by local and international regulations and country-specific radiation safety guidelines should be followed. For example, in Australia, the Australian Radiation Protection and Nuclear Safety Agency (ARPANSA) recommends “the ambient dose equivalent rate at a distance of 1 m from a patient who is undergoing treatment with a radioactive substance should not exceed 25 μSv/h at the time of the patient's discharge from hospital.” 17 18 Similarly, the U.S. Nuclear Regulatory Commission, Part 35 19 and the International Atomic Energy Agency Safety Reports Series 63 20 provide guidelines for safe discharge of radioactive patients to the public. The patient's discharge location and travel postdischarge are also important considerations which need to be determined prior to discharge. Depending on the location, radiation safety monitoring may be performed by the delegated radiation safety officer, or equivalent, providing the appropriate discharge radiation safety advice to the patient.

## Opportunities and Challenges

A large number of industry-driven trials strive to expand the use of targeted radiopharmaceutical therapies using tracers such as ^177^ Lu, ^225^ Ac, ^151^ Tb, ^89^ Zr, ^64^ Cu, and ^124^ I. The challenges posed by the availability of a large number of industry-driven trials include capacity issues, workforce shortages, appropriately trained staff, and equity of access to trials. These issues were recently addressed by a Lancet Oncology Commission, which addressed key issues impacting on implementation of theranostics at a global level, including workforce, radiopharmaceutical access, and regulatory issues. 5 21 22 23 The importance of adequate training for nuclear medicine physicians in theranostics has also been addressed through an international guidelines white paper. 24

## Conclusion

Clinical trials and theranostics in nuclear medicine are growing at a rapid pace. This requires a concerted effort to ensure that all personnel within the nuclear medicine department involved are appropriately trained for their roles. Clinical trials and theranostics require a thorough knowledge of the study protocol, intimate knowledge of the scanners and imaging protocols, including comprehensive training and credentialing, attention to detail, adhering to stipulated requirements, ability to communicate clearly with the patient and different stakeholders, and consistent compliance to GCP required for trial administration. Nuclear medicine departments should aim to have appropriately trained nuclear medicine physicians, nuclear medicine technologists, medical physicists, radiopharmaceutical scientists, nurses, study coordinators, and other staff in clinical trial and routine theranostics practice.
